# Position-dependent impact of hexafluoroleucine and trifluoroisoleucine on protease digestion

**DOI:** 10.3762/bjoc.13.279

**Published:** 2017-12-22

**Authors:** Susanne Huhmann, Anne-Katrin Stegemann, Kristin Folmert, Damian Klemczak, Johann Moschner, Michelle Kube, Beate Koksch

**Affiliations:** 1 Department of Chemistry and Biochemistry, Freie Universität Berlin, Takustraße 3, 14195 Berlin, Germany

**Keywords:** fluorinated amino acids, hexafluoroleucine, peptide drugs, protease stability, trifluoroisoleucine

## Abstract

Rapid digestion by proteases limits the application of peptides as therapeutics. One strategy to increase the proteolytic stability of peptides is the modification with fluorinated amino acids. This study presents a systematic investigation of the effects of fluorinated leucine and isoleucine derivatives on the proteolytic stability of a peptide that was designed to comprise substrate specificities of different proteases. Therefore, leucine, isoleucine, and their side-chain fluorinated variants were site-specifically incorporated at different positions of this peptide resulting in a library of 13 distinct peptides. The stability of these peptides towards proteolysis by α-chymotrypsin, pepsin, proteinase K, and elastase was studied, and this process was followed by an FL-RP-HPLC assay in combination with mass spectrometry. In a few cases, we observed an exceptional increase in proteolytic stability upon introduction of the fluorine substituents. The opposite phenomenon was observed in other cases, and this may be explained by specific interactions of fluorinated residues with the respective enzyme binding sites. Noteworthy is that 5,5,5-trifluoroisoleucine is able to significantly protect peptides from proteolysis by all enzymes included in this study when positioned N-terminal to the cleavage site. These results provide valuable information for the application of fluorinated amino acids in the design of proteolytically stable peptide-based pharmaceuticals.

## Introduction

Peptide-based drugs are promising pharmaceuticals since they offer several advantages including high selectivity, specificity, and efficacy for recognizing and binding to their targets [1–6]. However, their application as drugs is often limited due to low oral bioavailability and a short half-life attributable in part to proteases of the digestive system and blood plasma [1–8]. Efficient approaches to overcome these limitations have been developed including the incorporation of non-natural amino acids, such as D-amino acids, backbone-extended or chemically modified amino acids [1]. In this regard, the incorporation of fluorine into amino acids has become a promising strategy. Fluorine’s unique properties, namely low polarizability, a strong inductive effect, and high electronegativity, as well as its small size, result in strong, short C–F bonds and perturb the acidity and basicity of adjacent functional groups. Moreover, these changes may strongly influence hydrogen bonding and electrostatic interactions that are crucial for binding to receptors or, in context of protease stability, enzymes. Thus, when introduced in the form of fluorinated amino acids, this unique element can alter the biophysical, chemical and pharmaceutical properties of proteins and peptides including their interaction with proteases [9–10].

Several laboratories have focused on introducing highly fluorinated analogues of hydrophobic amino acids and have studied the effects on stability of the resulting proteins towards thermal and chemical denaturation [9,11–22]. These studies prompted further investigation into the extent to which fluorinated amino acids stabilize peptides and proteins against proteolytic degradation in particular. Meng and Kumar reported that the incorporation of 5,5,5,5’,5’,5’-hexafluoroleucine (HfLeu) into the antimicrobial peptides magainin 2 amide and buforin enhanced their resistance towards proteolytic degradation by trypsin [23]. They also introduced HfLeu into the glucagon-like-peptide-1 (GLP-1), which is an attractive lead compound for the treatment of diabetes mellitus type 2. Unfortunately, the clinical use of the wild-type peptide is severely hampered due to rapid digestion (≈2 min) by the serine protease dipeptidyl peptidase [24–26]. Satisfyingly, the fluorinated GLP-1 analogues displayed higher proteolytic stability against this enzyme [27].

Usually, the enhanced proteolytic stability of fluorinated peptides is explained by their greater hydrophobicity and altered secondary structure compared to the parent, non-fluorinated peptide. A further reason is the increased steric bulk of the fluorinated amino acid, meaning protection from protease degradation is a result of the steric occlusion of the peptide from the active site [23,28]. In contrast, the Marsh lab found that the introduction of HfLeu into the antimicrobial peptide MSI-78 only renders it more stable towards proteolysis by trypsin and chymotrypsin in the presence of liposomes [29]. In the absence of liposomes, the fluorinated variants were as rapidly degraded as the non-fluorinated control, suggesting that the incorporation of HfLeu is not the only factor that prevents the peptide from being digested by proteases [29]. Fluorinated aromatic amino acids were also investigated regarding their impact on peptide proteolysis. For instance, incorporation of monofluorinated phenylalanine variants into the histone acetyltransferase protein tGN5 resulted in destabilization in a chymotrypsin digestion assay [30]. Substitution of tryptophan, tyrosine, and phenylalanine residues in a glycosylation-deficient mutant of *Candida antarctica* lipase B, CalB N74D, by their monofluorinated analogues, left the resistance to proteolytic degradation by proteinase K unchanged [31]. Incorporation of α-fluoroalkyl-substituted amino acids can also lead to proteolytically stable peptides, and proteases can even be used to synthesize α-fluoroalkyl-substituted peptides [32–38].

These results indicate that the influence of fluorinated amino acids on the proteolytic stability of peptides and proteins remains difficult to predict. In an attempt to systematically study the influence of fluorinated amino acids on the proteolytic stability of peptides, a 10-amino acid peptide (FA) was previously designed in our group, comprising the substrate specificities of the proteases α-chymotrypsin and pepsin [39–40]. 2-Aminobutanoic acid (Abu) and its fluorinated analogues 2-amino-4,4-difluorobutanoic acid (DfeGly) and 2-amino-4,4,4-trifluorobutanoic acid (TfeGly) were individually incorporated at either the P2, the P1’ or the P2’ position [41] to give nine different analogues of FA. In prior studies, we observed that the introduction of fluorine atoms into the Abu side chain can significantly improve or dramatically reduce resistance to hydrolysis by different enzymes and human blood plasma, depending upon the fluorine content of the side chain, the position of the substitution relative to the cleavage site and the particular protease [39–40].

Here, we extend these studies to include highly fluorinated, sterically demanding HfLeu, and 5,5,5-trifluoroisoleucine (TfIle) and to investigate their effects on proteolytic stability towards the serine proteases α-chymotrypsin, elastase, and proteinase K, and the aspartate protease pepsin.

## Results and Discussion

### Peptide design and structure

**Figure 1:**
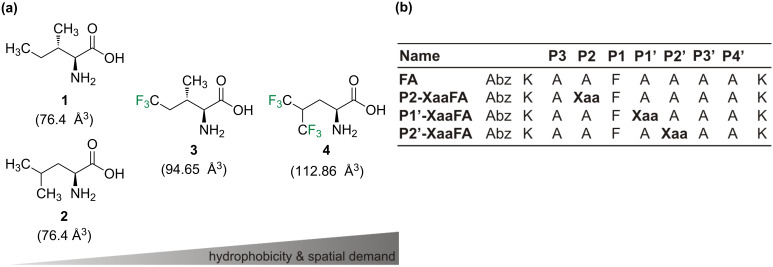
(a) Structures of isoleucine (**1**), leucine (**2**), and their fluorinated analogues 5,5,5*-*trifluoroisoleucine (**3**, TfIle) and 5,5,5,5’,5’,5’*-*hexafluoroleucine (**4**, HfLeu). The van der Waals volumes given in parentheses correspond to the side chains (starting at α-carbon), and were calculated according to Zhao et al. [43]; (b) Names and amino acid sequences of the studied peptides; the substitution positions are marked as Xaa; Xaa = Leu, HfLeu, Ile or TfIle. Positions are named according to Schechter and Berger nomenclature [41].

To elucidate the impact of fluorination on proteolytic stability we previously designed the peptide FA (Figure 1b) that comprises the substrate specificities of α-chymotrypsin and pepsin [39–40]. Consequently, the P1 position is occupied by a phenylalanine residue. Lysine residues were introduced at both ends of the peptide sequence to enhance solubility, and *o*-aminobenzoic acid (Abz) at the N-terminus serves as a fluorescence label. Alanine residues in positions P3, P3’, and P4’ act as spacers as the peptide binds in an extended conformation to the enzyme’s active site [42]. The positions P2, P1’ and P2’ at or adjacent to the cleavage site [41] carry the key residues for the recognition of the substrate by the protease and serve as substitution sites.

The alanines at P2, P1’ or P2’ positions were substituted individually with either TfIle or HfLeu (Figure 1a). Ile and Leu variants were also included in this study as non-fluorinated controls. This led to a library of 12 FA variants (Figure 1b).

Leu and Ile are larger and more hydrophobic than Ala. The fluorinated amino acids are even larger and more hydrophobic than their hydrocarbon analogues [44–45]. Furthermore, fluorine substitution has been shown to polarize neighboring C–H bonds (here the γ-hydrogens) that could affect noncovalent interactions [9,11]. Since the amino acids used here (Figure 1a) differ in their degree of fluorination, spatial demand and hydrophobicity, it is expected that they will have different impacts on the enzyme’s binding pocket, reflected by different behavior in the proteolysis assay.

### Determination of proteolytic stability

All peptides were incubated with the four different proteases and their proteolytic degradation was followed over a period of 24 h. Both, α-chymotrypsin [46–49] and pepsin [50–54] are well characterized digestive proteases. They are, together with trypsin, the main enzymes of the human digestive system. Elastase possesses a wide substrate specificity for non-aromatic, neutral side chains [55–56] and is found in the human pancreas and in blood serum. Proteinase K, an enzyme widely used for inactivation and degradation studies of proteins, was included here since it shows a broad substrate specificity and high activity and, thus, is able to digest numerous native proteins, even in the presence of detergents [57]. These four enzymes have different preferences at their subsites, thus providing a broad scope for our investigations.

The course of proteolytic digestion was characterized by an analytical HPLC-assay with fluorescence detection [39–40]. For quantification of substrate degradation, integration of the corresponding HPLC peak was conducted. Cleavage products were identified by ESI–ToF mass spectrometry (see Supporting Information File 1, Tables S4–S7). Figure S2 (Supporting Information File 1) shows the time course of all of the digestion experiments. A detailed description of the results for the individual enzymes is given in the following sections.

### Proteolytic stability towards α-chymotrypsin (EC 3.4.21.1)

α-Chymotrypsin is a serine endopeptidase with broad specificity. It preferably cleaves peptide bonds C-terminal to large hydrophobic residues such as phenylalanine, tyrosine, tryptophan, and leucine in the P1 position. Secondary hydrolysis also occurs at the carbonyl end of isoleucine, methionine, serine, threonine, valine, histidine, glycine, and alanine [47,58–60].

**Figure 2:**
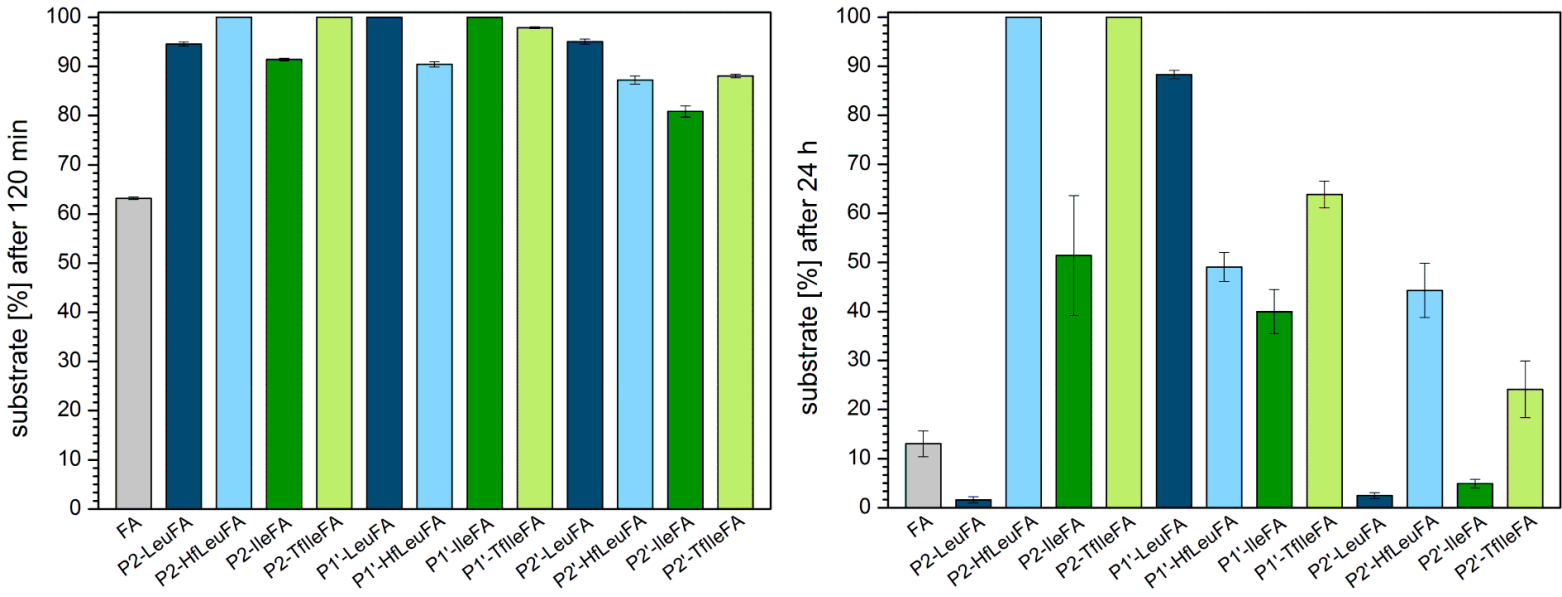
Percentage of substrate remaining after incubation for 120 min (left) and 24 h (right) with α*-*chymotrypsin in 10 mM phosphate buffer, pH 7.4, at 30 °C. The data shown represent the average of three independent measurements. Errors are derived from the standard deviation.

The S2 subsite of α-chymotrypsin generally prefers to accommodate hydrophobic residues [59,61]. We observed that the fluorinated P2 variants show a smaller amount of digestion after 120 min compared to their hydrocarbon analogues, while all variants are more stable than the control FA (Figure 2). After 24 h, all P2 peptides except for P2-LeuFA are still more stable than FA. Incorporation of Leu into P2 leads to complete proteolysis compared to FA, in which Ala occupies this position. However, incorporation of six fluorine substituents into Leu (resulting in HfLeu) results in an almost 100% gain in proteolytic stability. Ile is not as highly preferred in P2 as Leu, but also here the introduction of three fluorine substituents leads to a 50% gain in stability. P2-HfLeuFA and P2-TfIleFA are not digested at all, suggesting that HfLeu and TfIle are not favored within the S2 pocket of α-chymotrypsin.

P1’-substituted peptides are all more stable towards digestion than the control peptide FA, while Leu seems to provide the best protection from proteolysis. Here, introduction of fluorine makes the peptide prone to degradation. The opposite is true for Ile as TfIle leads to less efficient degradation. The S1’ subsite of α-chymotrypsin usually accommodates basic residues with long side chains [59,62–63]. Ile, as a branched amino acid, is obviously not well accommodated in this position for steric reasons. A further increase in side chain volume with TfIle exacerbates this effect. In the case of HfLeu, however, fluorine substituents seem to engage in favorable interactions with amino acid residues of the binding site, thus making P1’-HfLeuFA a better substrate than the non-fluorinated Leu peptide. Several such interactions are possible, as described in our previous work [39–40,64].

The S2’ subsite of α-chymotrypsin exhibits a hydrophobic character and thus prefers to accommodate hydrophobic residues [59,65]. However, the more hydrophobic peptides P2’-LeuFA, P2’-HfLeuFA, P2’-IleFA, P2’-TfIleFA are more stable against digestion by α-chymotrypsin compared to FA after 120 min of incubation. After 24 h, only the fluorinated analogues are less degraded than the control FA. Full length P2’-HfLeuFA and P2’-TfIleFA are present at percentages up to 44% and 24%, respectively, while substitution by Leu and Ile in P2’ position leads to accelerated proteolysis compared to FA. Thus, both HfLeu and TfIle have a protective effect towards proteolysis in this position.

**Figure 3:**
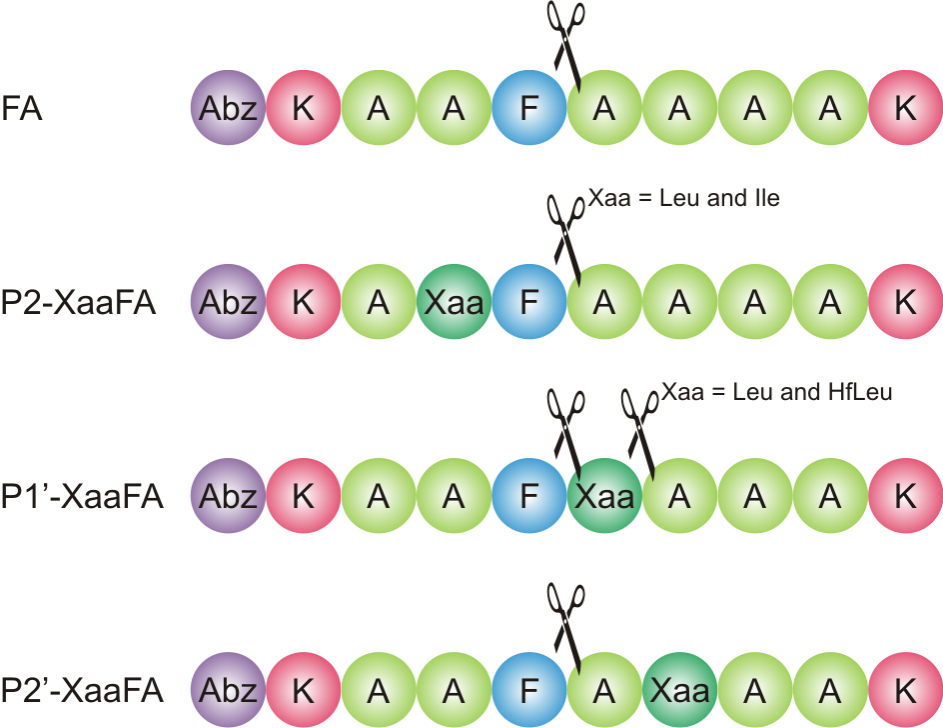
Cleavage positions observed in the digestion of library peptides with α-chymotrypsin.

ESI–ToF mass analysis confirms that the position P1 bearing Phe is the main cleavage site for α-chymotrypsin (Figure 3). Cleavage C-terminal to Leu and HfLeu in P1’-LeuFA and P1’-HfLeuFA is also observed (Figure 3, see Supporting Information File 1, Table S4), which means that the cleavage site was shifted towards the C-terminus by one residue. This is likely a consequence of α-chymotrypsin’s preference for not only aromatic residues but also bulky hydrophobic residues in the S1 pocket, thus, HfLeu is accepted by the P1 binding pocket of α-chymotrypsin.

In summary, the introduction of fluorinated Leu and Ile analogues into a α-chymotrypsin specific peptide sequence can improve proteolytic stability mainly at the P2 and P2’ positions, with the strongest effects observed for the P2 position.

### Proteolytic stability towards pepsin (EC 3.4.23.1)

Pepsin is an aspartic endopeptidase and one of the main digestive enzymes in humans. It exhibits specificity for hydrophobic, especially aromatic residues like Phe, Trp, and Tyr at the P1 and P1’ positions [50–54]. It has an extended active site that can bind at least seven residues [66–67], and peptide bond cleavage occurs N-terminal to the residue at position P1. The cleavage efficiency heavily depends upon the identity of this amino acid, with Phe and Leu being the most favored residues. At the P1’ position aromatic amino acid residues are preferred, however, the influence of the P1’ position on proteolytic cleavage is not as significant [68]. Pepsin typically does not cleave at Val, Ala, or Gly linkages [60].

**Figure 4:**
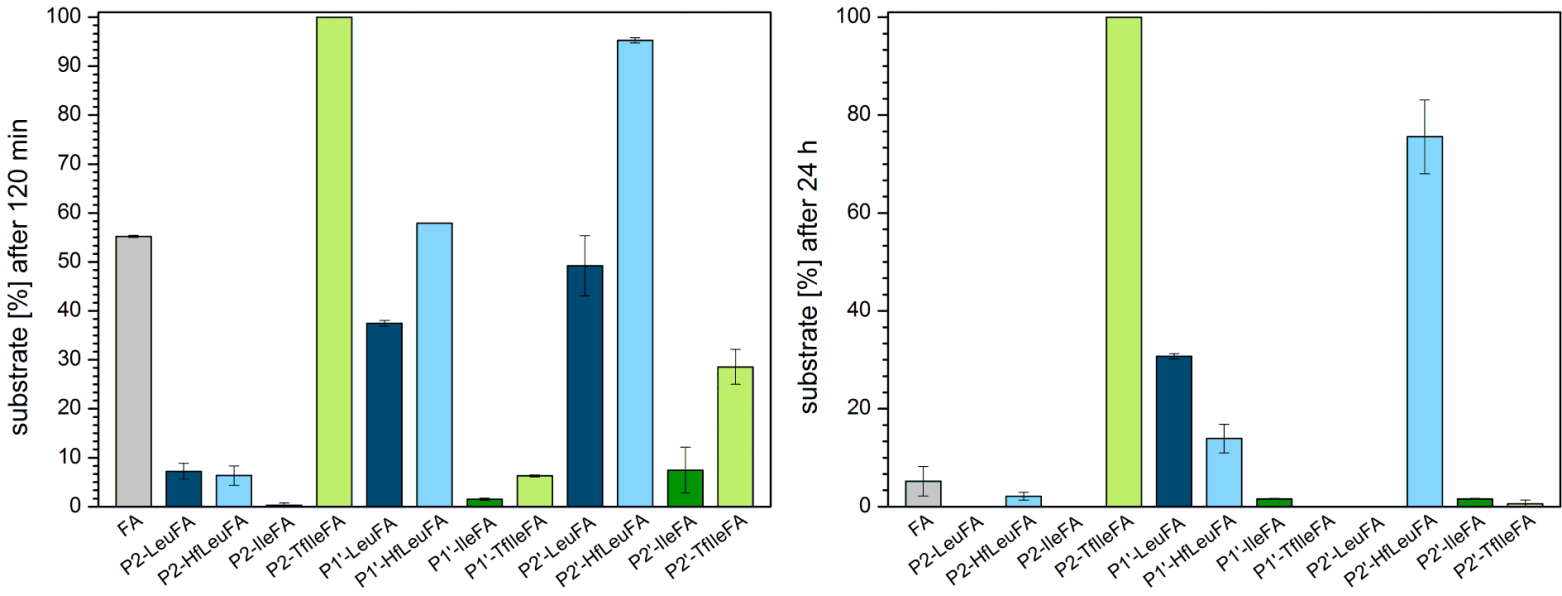
Percentage of substrate remaining after incubation for 120 min (left) and 24 h (right) with pepsin in 10 mM acetate buffer, pH 4.0, at 30 °C. The data shown represent the average of three independent measurements. Errors are derived from the standard deviation.

The S2 subsite of pepsin preferentially accommodates hydrophobic residues such as Leu, Ala or norleucine as well as the β-branched species Ile and Val, but can also bind charged residues [69–70]. Except for P2-TfIleFA, we observed that the P2-modified peptides are degraded more rapidly than the control peptide FA and that these peptides are almost or completely degraded after 120 min (Figure 4). For example, whereas after 24 h FA is also almost completely degraded, P2-TfIleFA is still detected at a level of 100%. Incorporation of Leu or Ile leads to complete proteolysis. Remarkably, the introduction of six fluorine atoms into Leu doesn’t change this behavior. In sharp contrast yet equally remarkable, the incorporation of three fluorine substituents into Ile results in a 100% gain in proteolytic stability. These results indicate that Leu, HfLeu, as well as Ile are well accommodated in the S2 subsite of pepsin. In contrast, TfIle, although smaller than HfLeu [44], doesn’t appear to fit well into the S2 pocket of pepsin.

To compare the P1’ substituted peptides, only P1’-HfLeuFA shows the same persistence after 120 min as the control peptide FA, while all other sequences are digested faster. Here, the introduction of fluorine into Leu seems to stabilize the peptide by about 20%. Interestingly, after 24 h this trend is reversed, and the P1’-HfLeuFA peptide is destabilized to an amount of 17% compared to the hydrocarbon analogue, but both peptides are somewhat more stable than the control FA. The incorporation of TfIle into this position doesn’t show a significant impact. Although the fluorine substituents slow down the digestion process (see Supporting Information File 1, Figure S2b), the TfIle containing peptide as well as its hydrocarbon analogue are fully digested after 24 h. The S1’ subsite has hydrophobic character and thus prefers to accommodate hydrophobic or aromatic residues [71]. Ile and TfIle are obviously well accommodated in this position, while Leu and HfLeu are not.

The S2’ subsite of pepsin favors hydrophobic amino acids, but also accepts charged polar amino acids like Glu and Thr [52,72]. After 120 min peptides P2’-LeuFA, P2’-IleFA and P2’-TfIleFA are degraded faster than the control FA. Instead, P2’-HfLeuFA is only digested up to 5%. This effect is even more pronounced after 24 h. While all other P1’ substituted peptides, along with the control peptide FA, are almost or completely digested, P2’-HfLeuFA is still present to about 76%. In this case fluorination leads to protection against proteolysis by pepsin. Hfleu is obviously not well accommodated in this position. As already observed for position P1’, the introduction of three fluorine atoms into Ile slows down proteolysis, although both peptides are completely digested after 24 h.

**Figure 5:**
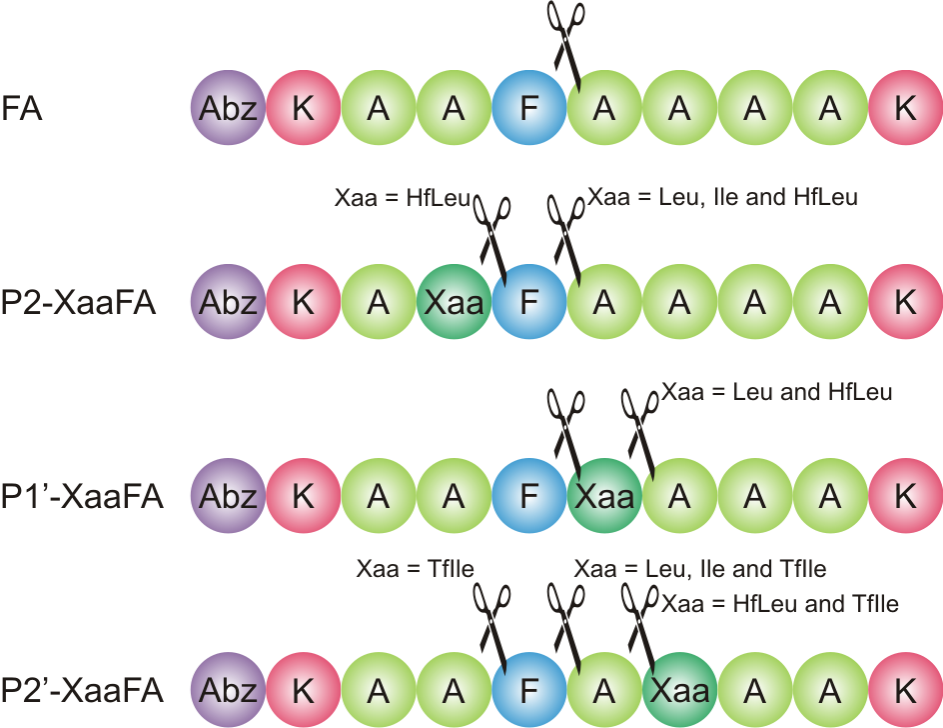
Cleavage positions for digestion of the different peptides with pepsin.

For almost all peptides of our library, we observed the expected cleavage pattern with Phe in the P1 position (Figure 5, see Supporting Information File 1, Table S5). Only P2’-HfLeuFA is not hydrolyzed at the designed cleavage site, instead cleavage occurs exclusively N-terminal to the HfLeu residue, thus demonstrating that HfLeu occupies the P1’ position. In the case of P2’-TfIle we found two further peptide bonds that are cleaved by pepsin, namely N-terminal cleavage to TfIle and to Phe. These findings indicate that the S1’ subsite accommodates bulky hydrophobic residues more readily than does the S2’ site of pepsin. For P1’-LeuFA and P1’-HfLeuFA we found an additional cleavage site at which the peptide bonds Leu^P1’^-Ala^P2’^, and HfLeu^P1’^-Ala^P2’^ are hydrolyzed, respectively, which means that the cleavage site was shifted towards the C-terminus by one residue. This cleavage pattern was also detected for α-chymotrypsin before, and indicates that HfLeu is well accepted by pepsin in its S1 binding site. Furthermore, we identified a second cleavage site for P2-HfLeuFA at which the peptide bond N-terminal to Phe is proteolytically cleaved as well. This means that the cleavage site is shifted such that HfLeu occupies the P1 and Phe the P1’ position. However, this perfectly matches the specificity of pepsin that prefers bulky hydrophobic and aromatic amino acids both up- and downstream of the scissile bond.

In summary, the introduction of fluorinated Leu into a pepsin specific peptide sequence can improve the proteolytic stability at the P2’ position, whereas the incorporation of a fluorinated Ile into the P2 position shows the strongest effect in protection from proteolysis.

### Proteolytic stability towards elastase (EC 3.4.21.36)

Elastase is a serine endopeptidase, and has a wide specificity for non-aromatic uncharged side chains. It preferentially cleaves peptide bonds C-terminal to small uncharged non-aromatic amino acid residues such as glycine, alanine and serine, but also valine, leucine, isoleucine [56,73]. Its binding site extends over eight subsites (S5 to S1, and S1’ to S3’) [74].

**Figure 6:**
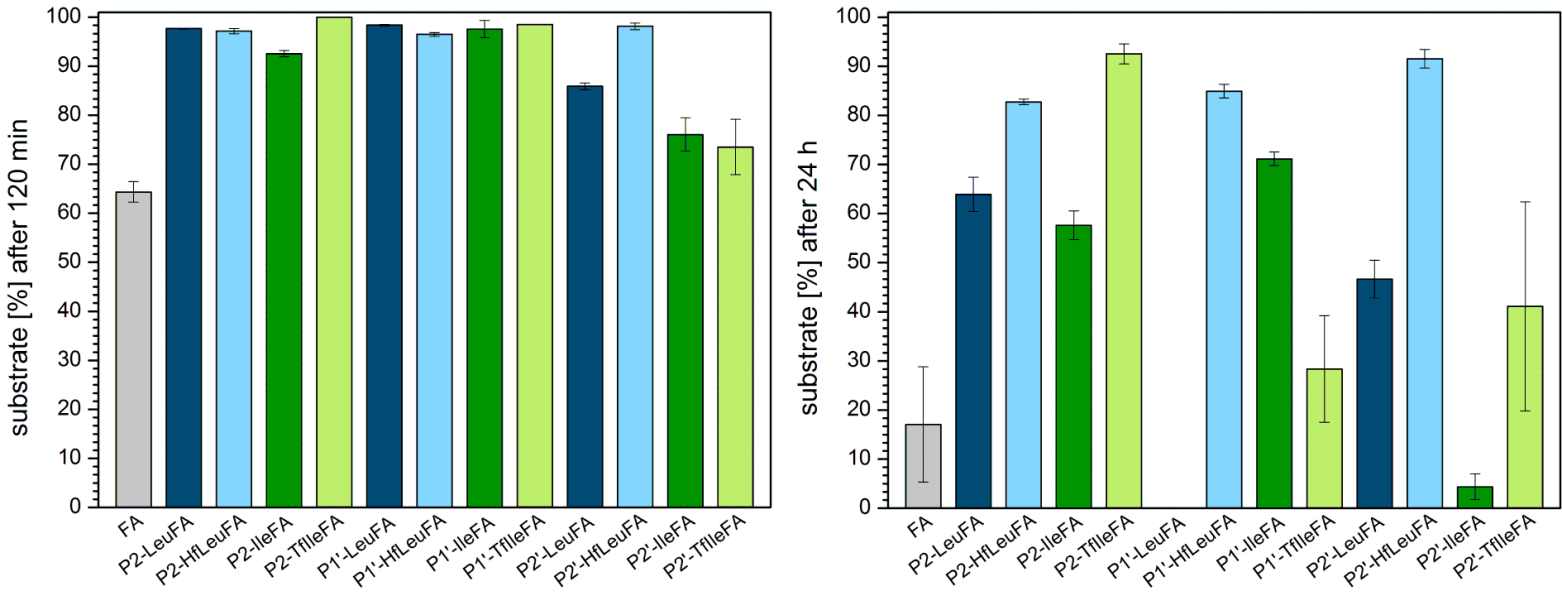
Percentage of substrate remaining after incubation for 120 min (left) and 24 h (right) with elastase in 100 mM Tris/HCl buffer, pH 8.4, at 37 °C. The data shown represent the mean of three independent measurements. Errors are derived from the standard deviation.

The fact that in this study larger and more hydrophobic amino acids [44–45] were introduced may explain why degradation of most of the variants during the first 120 min of incubation with elastase is hardly observed (Figure 6). Only P2’-LeuFA, P2’-IleFA, and P2’-TfIleFA were somewhat digested during this time, however, all of the modified peptides are more stable than the control FA.

After 24 h all P2 peptide variants are more stable than the control FA (Figure 6), while TfIle provides the best protection from elastase digestion. Leu and Ile are not quite as preferred in P2 position as is Ala. Fluorination of Leu leads to an increase in stability of around 19%. With 35% this effect is even higher when three fluorine atoms are introduced into Ile.

Modification of the P1’ postion renders P1’-HfLeuFA and P1’-IleFA more stable than the control peptide FA, while P1’-TfIleFA is comparably stable. Incorporation of Leu into P1’ leads to complete digestion. However, introducing six fluorine atoms into Leu results in an 85% gain in stability. The opposite is observed for Ile, where TfIle accelerates enzymatic degradation.

Except for P2’-IleFA, all P2’ modified variants are more stable compared to the control peptide FA after 24 h. Leu is not as preferred in this position as Ala. Introduction of fluorine strengthens this effect and effectively doubles the stability. In contrast, introduction of Ile leads to almost complete proteolysis. However, substitution by TfIle slows down the degradation rate and results in a stabilization of around 37%. In P2’, fluorination shows in both cases a protective effect towards hydrolysis by elastase.

**Figure 7:**
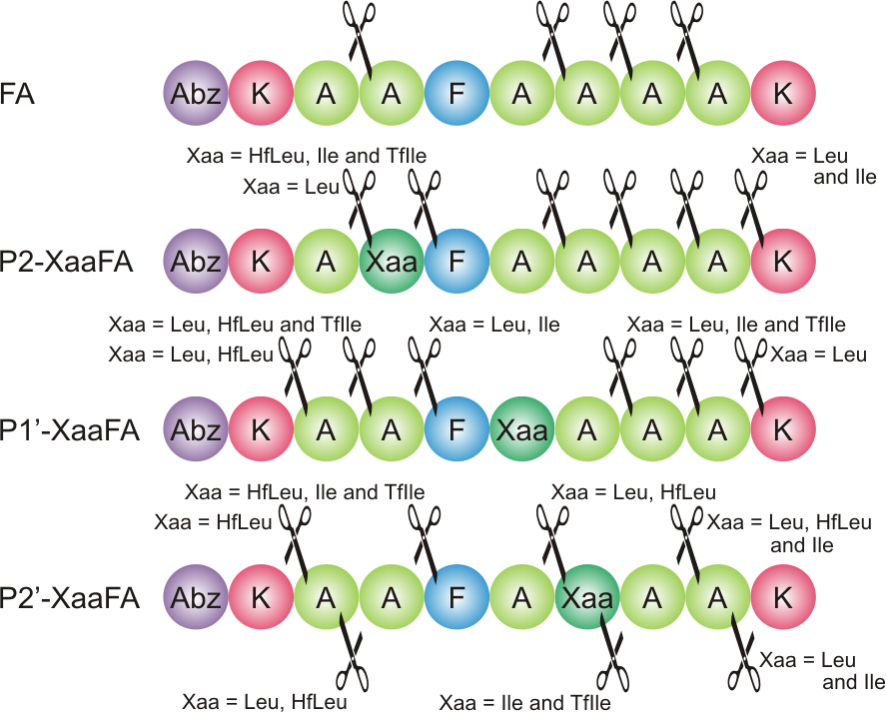
Cleavage positions for digestion of the different peptides with elastase.

Elastase preferably hydrolyses peptide bonds C-terminal to uncharged non-aromatic amino acids and mainly between Ala–Ala and Ala–Gly bonds [56,73]. Since Ala is the main residue present in the peptides studied here, we observed various cleavage products in the ESI–ToF analysis (Figure 7, see Supporting Information File 1, Table S6). For none of the peptides were fragments with Phe in the P1 position observed. Since elastase has a constricted S1 pocket, the binding of aromatic amino acids at P1 is deleterious [75]. Here, we also observed that Leu appears to never occupy the P1 position, although it is known to occupy this position in other substrates [73]. Interestingly, the larger fluorinated variant was found in the P1 position in one case, while Ile and its fluorinated analogue occupy this position in two of the three peptide analogues.

The S2’ subsite of elastase has a marked specificity for Ala, and can accommodate bulkier residues only with some difficulty [74]. Thus, we did not find the fluorinated amino acids HfLeu and TfIle binding to the S2’ subsite of the enzyme as expected, whereas for the Leu and Ile variants this was observed only in one case each.

The S1’ subsite usually prefers Lys residues, and to a lesser extent Ala or Glu [74,76]. Indeed, we found a fragment cleaved off corresponding to a Lys in P1’, but primarily detected fragments with Ala in P1’ and also Phe that was even more favored than Lys. We observed that Ile as well as its fluorinated analogue TfIle are not accommodated in this subsite, probably due to their β-branched topology.

The S3’ pocket in elastase is known to have a high aromatic specificity [74]. Interestingly, in our cases Phe in P3’ was less favored. Instead, mainly Lys occupied this position.

Ala is favored in P2. Its carboxyl group can form a hydrogen bond with the amide nitrogen of Gly193 in the S2 pocket, and Ala’s methyl group faces the solvent [76].

Occupation of the S4 subsite is important for efficient catalysis [76–77]. Thus elastase might not easily split the first three bonds at the amino terminus of a peptide chain, since interactions of a residue with S4 is necessary [77]. Indeed, we only observed a low amount of cleavage proximal to the N-terminus, while most of the hydrolysis occurred at the C-terminal end of the peptides. The S3 subsite seems to accommodate bulkier hydrophobic amino acids well, as we observed cleavage products containing Ile and Leu in P3 position for all the peptides modified with these residues, as well as their fluorinated analogues for two of the three substituted peptides each.

In summary, introduction of HfLeu in different positions of a peptide can enhance the proteolytic stability up to 85% compared to the corresponding Leu analogues. Replacing Ile with TfIle can increase the stability against elastase as well, although not as efficiently as HfLeu.

### Proteolytic stability towards proteinase K (EC 3.4.21.64)

Proteinase K is a non-specific serine endopeptidase and the main proteolytic enzyme produced by the fungus *Tritirachium album Limber* [78]. It has a broad substrate specificity, cleaving peptide bonds C-terminal to a number of amino acids, however, prefers aromatic or aliphatic hydrophobic amino acids in position P1 [57,78]. Furthermore, Ala is favored in position P2 and enhances cleavage efficiency [79–80]. Proteinase K possesses a very high proteolytic activity [79]. Its active center contains an extended binding region consisting of several subsites, at least four or five subsites on the N-terminal side of the scissile bond (S1 to S4/S5) and three subsites C-terminal to the scissile bond (S1’ to S3’) [81–83]. The “bottom” of substrate recognition site is predominantly hydrophobic and there is evidence that not the sequence of the substrate is of importance in the recognition but only the volume of the side chains [84].

**Figure 8:**
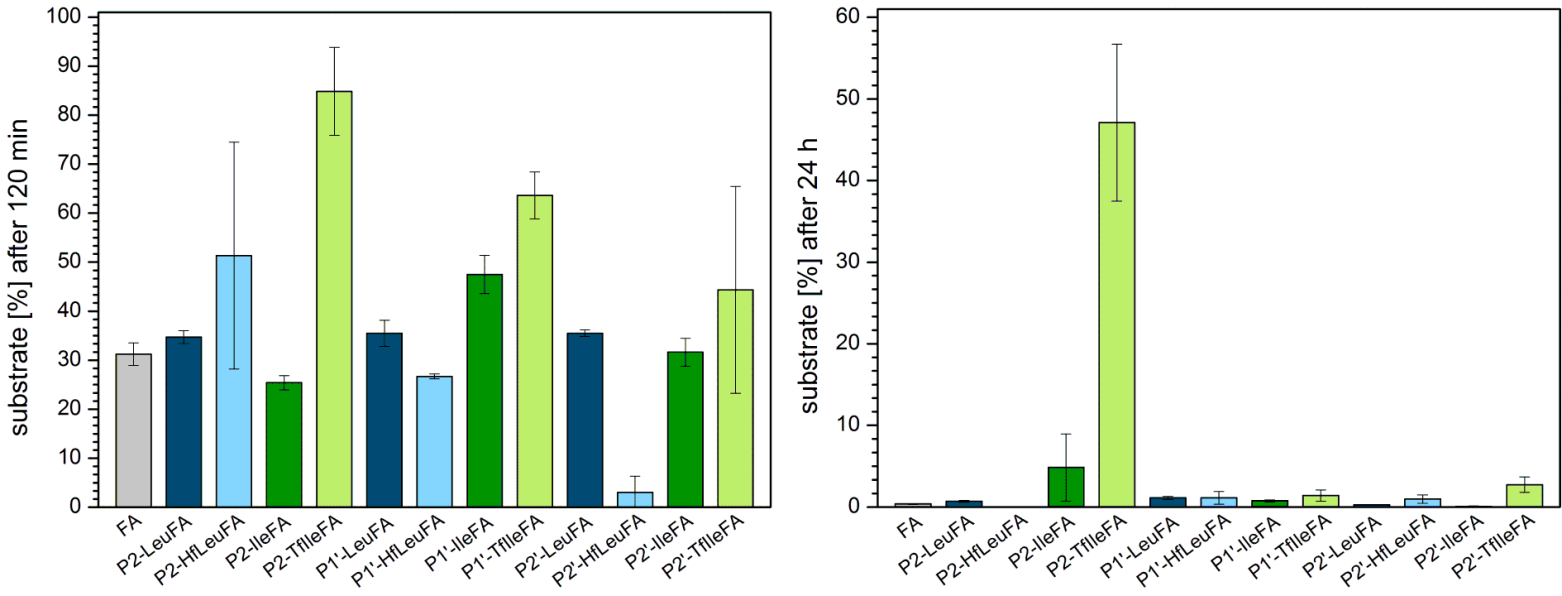
Percentage of substrate remaining after incubation for 120 min (left) and 24 h (right) with proteinase K in 50 mM Tris/HCl buffer, containing 10 mM CaCl_2_ pH 7.5, at 37 °C. The data shown represent the mean of three independent measurements. Errors are derived from the standard deviation.

Substitution of Ala in position P2 with Ile and Leu leads to a greater or comparable amount of degradation after 120 min. Introducing fluorine atoms in both cases slows down the digestion process, most pronounced for P2-TfIleFA with a gain of 60% in stability compared to its non-fluorinated analogue. Ile is not preferred to the extent that Leu is, and the introduction of fluorine enhances this effect. While all other peptides are almost completely or entirely degraded after 24 h, P2-TfIleFA is the only peptide that is still left after 24 h of incubation (Figure 8).

Introduction of Leu at the P1’ position leads to an amount of digestion comparable to FA after 120 min. Fluorination of the Leu side chain leads to a small acceleration in digestion. Ile at this position is not as preferred as is Leu and this enhances the stability to a small extent compared to FA. Introducing three fluorine atoms at the Ile side chains strengthens the stability against proteinase K even further.

As already observed for the other two Leu containing peptides, also substitution of Ala at position P2’ with Leu does not change the resistance against proteinase K significantly. Interestingly, when six fluorine atoms are introduced, the digestion process is faster and P2’-HfLeuFA is almost completely degraded after 120 min. The opposite is observed for the fluorination of Ile. While P2’-IleFA is as stable as FA, P2’-TfIleFA shows a small gain in stability of around 12%.

Thus, in the case of P1’ as well as P2’ peptide variants, only fluorination of Ile leads to a slower digestion by proteinase K, while introducing even more fluorine atoms into the Leu side chain leads to more rapid hydrolysis compared to the non-fluorinated analogues. Ile seems in all investigated cases not as preferred as Leu, since less efficient digestion is observed. Introduction of three fluorine atoms even enhances this protective effect.

**Figure 9:**
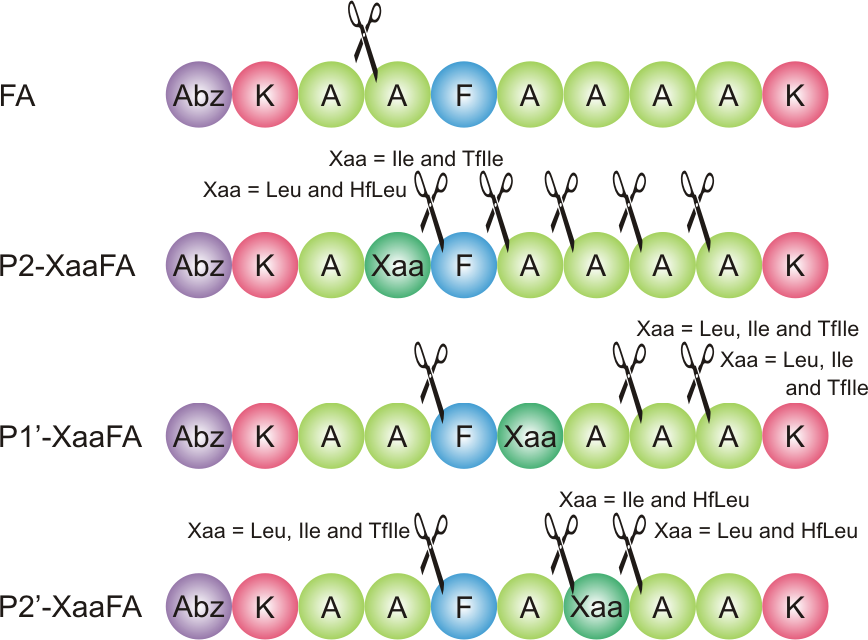
Cleavage positions found with proteinase K.

Based on the wide substrate specificity of proteinase K and its preference for alanine, and since our studied peptides have a high number of alanine residues present, there are multiple cleavage sites possible in addition to the designed site between Phe^P1^ and Xaa^P1’^. Indeed, multiple cleavage patterns are observed, especially cleavage C-terminal to Ala (Figure 9, see Supporting Information File 1, Table S7). Thus, Ala mainly occupies the S1 subsite, but is also found to bind to the S2 site to a greater extent. Ala is most effective in P2 [80] as the S2 subsite is a small and narrow cleft, which limits the possibilities for effective side chain substitutions [79]. However, Ile and TfIle are well accepted here. A negative or positive charge at S2 is not preferred and hampers the formation of the enzyme substrate complex [82]. Thus, it can be concluded that Lys is poorly accepted at this position. HfLeu, the most sterically bulky amino acid investigated here, is not observed to occupy the S2 subsite. Instead, HfLeu is mainly found to bind to the S1 pocket. Leu is also found to occupy the S1 site of proteinase K, which is large and has mainly hydrophobic character [82–83,85]. It does not impose too strong steric limitations on the amino acid side chain but prefers hydrophobic and aromatic residues, with a specificity for Ala [78,81,83,86]. Charged side chains of Glu and Lys are very poorly accepted, as are β-branched functional groups, because the entrance to the S1 subsite is too narrow to allow their passage [79]. Thus, Lys is not observed to occupy the S1 subsite. Neither Ile nor TfIle can be accommodated by the S1 pocket due to their β-branching. Phe is found in S1 in only two cases in our study, and mainly occupies the S3 and S1’ pockets. The S3 pocket has a wide specificity due to its location at the protein surface, but exhibits preference for aromatic side chains in P3 (Trp, Phe) [79]. S1’ shows a slight preference for smaller residues like Ala and Gly, but also bulkier residues such as Phe and Leu are hydrolyzed to a significant extent [81]. In this study Leu apparently does not bind to the S1’ site at all, and this is also true of TfIle. Additionally, Lys is not well accommodated here. Phe is also found to occupy S4 to a great extent, and this subsite is known to have an affinity for aromatic groups, especially a marked preference for Phe [79]. S4–P4 interactions are primarily hydrophobic in nature [79], which might explain why we observed that Lys is only poorly accepted in this position. The S3 subsite cannot be defined as a “cleft” or “pocket” [79]. The P3 residue of the peptide substrate lies on the protein surface and the side chain of P3 should be directed toward the solvent [79]. This arrangement might explain the broad specificity of S3 [79]. We observed that all the residues used in this study can occupy the P3 position, mainly Phe and Lys. Leu, Ile and TfIle are also found to a great extent in P3.

In summary, fluorination of an Ile residue N-terminal to the cleavage site can help to protect a peptide against proteolysis by proteinase K. Due to its broad specificity and high activity, proteinase K typically digests peptides quickly [57]. This was also observed in this work in experiments in which all peptides, except for P2-TfIleFA, a remarkably stable species, were completely degraded after an incubation time of 24 h.

## Conclusion

**Figure 10:**
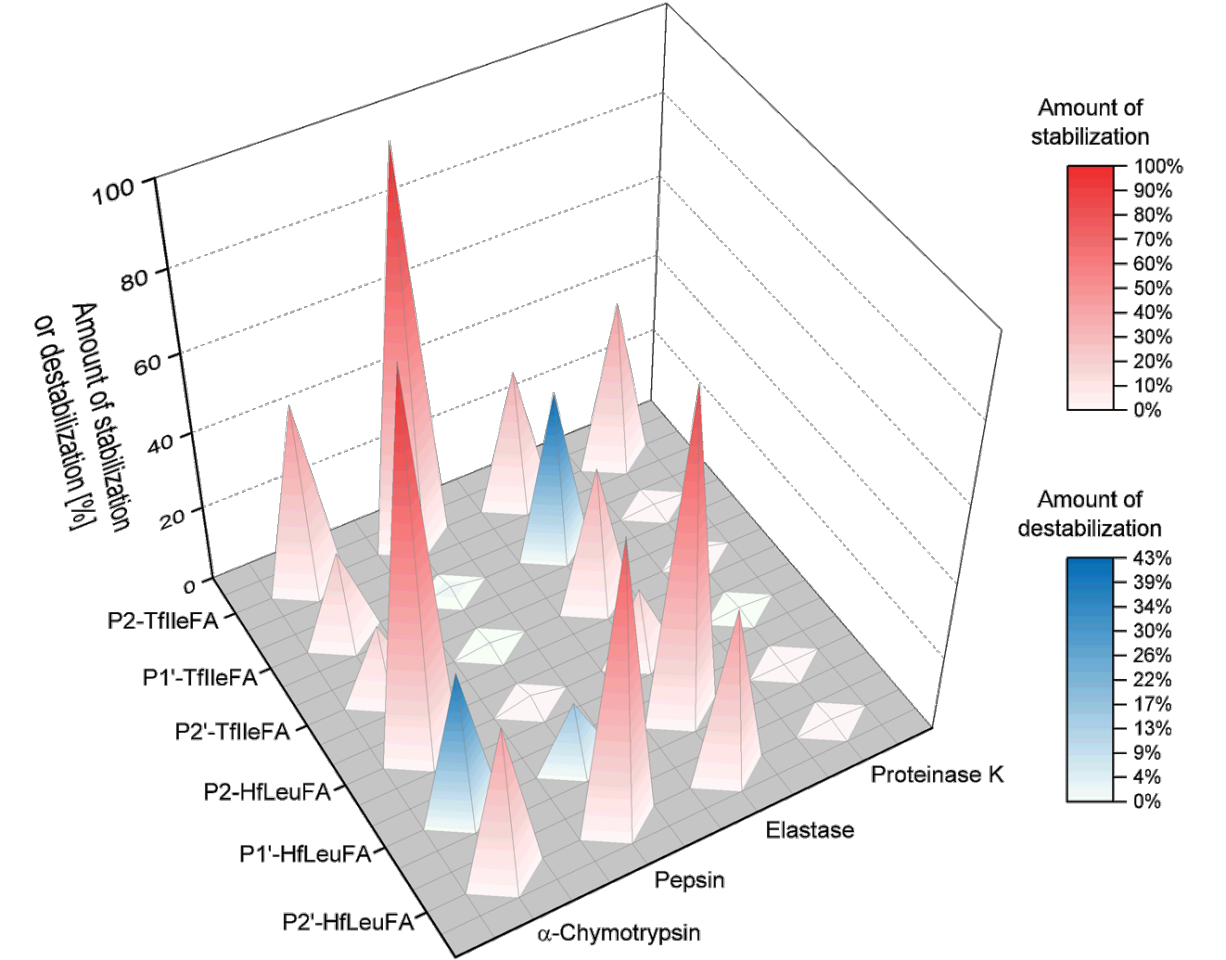
Dimension of stabilization or destabilization upon TfIle or HfLeu incorporation compared to the non-fluorinated analogues containing Ile or Leu, respectively, for all four different enzymes studied here and measured after 24 h of incubation.

The bulky side-chain fluorinated amino acids HfLeu and TfIle have the power to significantly stabilize peptides against proteolytic degradation. The impact of their incorporation on the proteolytic stability of peptides does not follow a general trend but rather depends on a combination of factors including the nature of the fluorinated amino acid, the substitution position relative to the cleavage site and the studied protease. Also, in contrast to proteolytic studies published before [23,27–28], the expectation of a general increase in proteolytic stability as a result of steric occlusion of the peptide from the active site upon incorporation of sterically demanding fluorinated amino acids could not be verified based on the results of our current study. We found a significant stabilization towards proteolysis in 13 of a total of 24 peptides of the library studied here upon introduction of either HfLeu or TfIle (Figure 10). However, we observed that even these sterically demanding fluorinated amino acids show in some cases favorable interactions with the enzymes binding sites resulting in a more rapid digestion as the non-fluorinated control.

The introduction of fluorinated Leu and Ile analogues into P2 and P2’ position improved the proteolytic stability towards α-chymotrypsin. When introduced in the P1’ position a stabilization was still observed for TfIle, while incorporation of HfLeu made the peptide more prone to proteolytic digestion compared to the non-fluorinated control. Incorporation of HfLeu had a significantly stabilizing effect towards hydrolysis by pepsin only in P2’ position, while TfIle develops a protective effect only when incorporated in P2 position.

As both, elastase and proteinase K possess a broad specificity, preferring C-terminal cleavage to Ala, we observed here a rather unspecific cleavage pattern for both enzymes with multiple cleavage products, in which the intended designed cleavage site with Phe in P1 position wasn’t affected. However, we observed that the introduction of HfLeu has a general protective effect against degradation by elastase, whereas the effect of TfIle depends on the substitution position. Although the introduction of fluorine substituents generally affected the rate of hydrolysis by proteinase K, only fluorination of an Ile residue N-terminal to the cleavage site effectively protected the peptide from digestion. Particularly noteworthy is the effect of fluorination of the Ile side chain in P2 position. The P2-TfIleFA peptide was the most resistant substrate towards proteolysis by all four proteases applied in this study. In contrast, destabilization due to fluorination was only observed when TfIle and HfLeu were incorporated into the P1’ position.

In future studies, we will focus on a more precise characterization of the interaction of fluorinated substrates with proteolytic enzymes to which multiple factors contribute. The steric demand or conformation of the side chain, hydrophobicity, fluorine induced polarity and significant p*K*_a_-value changes of neighboring groups [9–10] can lead to fluorine-specific interactions between substrate and enzyme binding sites as well as to an exclusion of the cleavage-relevant peptide bonds from the active site.

Furthermore, our investigations show that fluorine’s impact on proteolytic stability needs to be investigated always case-by-case as there is no general trend to be concluded. Nevertheless, the results of this current study provide valuable knowledge on how bulky fluorinated amino acids can help to increase the proteolytic stability of peptides, and show that upon smart design, these fluorinated amino acids can be used to engineer peptide drug candidates.

## Experimental

### Materials

Fmoc-L-amino acids were purchased from ORPEGEN Peptide Chemicals GmbH (Heidelberg, Germany). Fmoc-Lys(Boc)Wang resin was from Novabiochem (Merck Chemicals GmbH, Darmstadt, Germany)*.* All solvents were used from VWR (Darmstadt, Germany) without further purification. All other chemicals were bought from Acros (Geel, Belgium), abcr GmbH (Karlsruhe, Germany), fluorochem (Hadfield, United Kingdom), VWR (Darmstadt, Germany) or Merck (Darmstadt, Germany) at highest commercially available purity and used as such. A detailed synthetic strategy for Fmoc-TfIle-OH is described in literature [44]. For the synthesis of Fmoc-HfLeu-OH see Supporting Information File 1.

### Peptide synthesis, purification and characterization

Peptides were synthesized manually in a 0.05 mmol scale on a solid support by means of an Fmoc/*tert*-butyl protecting group strategy on a preloaded Fmoc-Lys(Boc)Wang resin (0.57 mmol/g loading) using 10 mL polypropylene reactors. HfLeu containing peptides were synthesized with an Activo-P11 automated peptide synthesizer (Activotec, Cambridge, United Kingdom). Couplings of non-fluorinated amino acids were performed in dimethylformamide (DMF) with the Fmoc-L-amino acid, 1-hydroxybenzotriazole (HOBt) and *N,N’*-diisocarbodiimide (DIC) in an eight-fold excess with respect to the resin amount. In order to ensure completion of the reaction the couplings were performed twice for 1 h each. The fluorinated amino acids and coupling reagents 1-hydroxy-7-azabenzotriazole (HOAt)/DIC were used in 1.2-fold excess, and the coupling was carried out manually one time overnight. In the case of an insufficient coupling, the coupling was repeated for 3 h with 0.5 equivalents. Prior to the Fmoc deprotection of the fluorinated amino acids, free N-termini were capped by adding a mixture of acetic anhydride (Ac_2_O, 10% (v/v)) and *N,N*-diisopropylethylamine (DIPEA, 10% (v/v)) in DMF (3 × 10 min). Fmoc deprotection was achieved by treatment with 20% (v/v) piperidine in DMF (3 × 10 min). All peptides were N-terminally labeled with *o*-aminobenzoic acid (Abz) to enable photometric detection. The resin was washed between each step with DMF and dicholoromethane (DCM, 3 × 2 mL each). After the synthesis, the peptides were cleaved from the resin by treatment with a solution (2 mL) containing triisopropylsilane (TIS, 10% (v/v)), water (1% (v/v)), and trifluoroacetic acid (TFA) (89% (v/v)) for 3 h. The resin was washed twice with TFA (1 mL) and DCM (1 mL) and excess solvent was removed by evaporation. The crude peptide was precipitated with ice-cold diethyl ether (80 mL), and after centrifugation dried by lyophilization. Purification of the synthesized peptides was performed on a LaPrepΣ low-pressure HPLC system (VWR, Darmstadt, Germany) using a Kinetex RP-C18 endcapped HPLC column (5 µM, 100 Å, 250 × 21.2 mm, Phenomenex^®^, USA). A Security GuardTM PREP Cartridge Holder Kit (21.20 mm, ID, Phenomenex^®^, USA) served as pre-column. As eluents deionized water (Milli-Q Advantage^®^ A10 Ultrapure Water Purification System, Millipore^®^, Billerica, MA, USA) and acetonitrile (ACN), both containing 0.1% (v/v) TFA were used. HPLC runs were performed starting with an isocratic gradient of 5% ACN over 5 min, flow rate: 10 mL/min, continuing with a linear gradient of 5–70% ACN over 25 min, flow rate: 20.0 mL/min. UV-detection occurred at 220 nm. Data analysis was performed with an EZChrom Elite-Software (Version 3.3.2 SP2, Agilent Technologies, Santa Clara, CA, USA). The fractions containing pure peptide were combined, reduced in vacuo and lyophilized to give the peptides as a white powder. The purity of the peptides was controlled by analytical HPLC (LUNA^TM^ C8 (2) column, 5 μm, 250 × 4.6 mm, Phenomenex^®^, Torrance, CA, USA), and the products were identified by high-resolution ESI–ToF–MS (see Supporting Information File 1).

### Protease digestion assay

All peptides employed in the degradation studies were used as the TFA salts obtained after lyophilization. Stock solutions of α-chymotrypsin (from bovine pancreas, EC 3.4.21.1, ≥40.0 units/mg of protein, Sigma Aldrich, St. Louis, MO, USA), and pepsin (from porcine stomach mucosa, EC 3.4.23.1, ≥250 units/mg of protein, Sigma Aldrich, St. Louis, MO, USA) were prepared at concentrations of 1 mg/mL in phosphate buffer (10 mM, pH 7.4), or in acetate buffer (10 mM, pH 4.0), respectively. For proteinase K (from tritirachium album, EC 3.4.21.64, ≥30 units/mg of protein, Sigma Aldrich, St. Louis, MO, USA) and elastase (from porcine pancreas, EC 3.4.21.36, 6.2 units/mg of protein, Sigma Aldrich, St. Louis, MO, USA) stock solutions were prepared also at concentrations of 1 mg/mL in tris/HCl (50 mM) + CaCl_2_ (10 mM) buffer (pH 7.5), or in tris/HCl buffer (100 mM, pH 8.4), respectively. Peptides (0.002 mmol) were prepared as stocks in DMSO (100 µL) and incubated with the respective enzyme at 30 °C (for α-chymotrypsin and pepsin) or 37 °C (for proteinase K and elastase) with shaking at 300 rpm in a thermomixer over a period of 24 h. The reaction mixture consisted of DMSO (15 µL), corresponding buffer (25 µL), peptide solution (5 µL) and the corresponding enzyme solution (5 µL). The concentration of enzyme was optimized so that the hydrolysis of the control peptide FA was about 40% after 120 min. Aliquots of 5 µL were taken at fixed time points (0, 15, 30, 60, 90, 120 min as well as 3 h and 24 h) and either quenched with ACN containing 0.1% (v/v) TFA (95 µL), in the case of α-chymotrypsin, proteinase K and elastase, or 2% aqueous ammonia (95 µL), in the case of pepsin. All samples were subjected to analytical HPLC on a LaChrom-ELITE-HPLC-System equipped with a fluorescence detector (VWR International Hitachi, Darmstadt, Germany). A monolithic reversed-phase C8 Chromolith^®^ Performance HPLC column (100 × 4.6 mm, Merck KGaA, Darmstadt, Germany) was used to resolve and quantify the products of digestion. The used system and gradients are described in detail in Supporting Information File 1. Detection based on the Abz label was carried out using a fluorescence detector with λ_ex_ = 320 nm and λ_em_ = 420 nm. In all cases, the peaks corresponding to the starting materials (full-length peptides) or the N-terminal fragments (products) were integrated and used to determine the velocity of the reaction (see Supporting Information File 1). The FA peptide was used as a reference. Each fragment cleaved from the full-length peptide was identified by ESI–ToF mass analysis on an Agilent 6220 ESI–ToF–MS spectrometer (Agilent Technologies, Santa Clara, CA, USA, see Supporting Information File 1). All experiments were performed in triplicate.

## Supporting Information

File 1 Characterization and identification of synthesized peptides, characterization of the enzymatic digestion reactions, and identification of proteolytic cleavage products, HPLC methods, and synthesis protocol for Fmoc-HfLeu-OH.
