# Supplementary material for: Position-dependent impact of hexafluoroleucine and trifluoroisoleucine on protease digestion
Source: Beilstein J Org Chem. 2017 Dec 22;13:2869–82. doi: 10.3762/bjoc.13.279 (PMC5753150; doi:10.3762/bjoc.13.279)
Supplement: File 1 — Characterization and identification of synthesized peptides, characterization of the enzymatic digestion reactions, and identification of proteolytic cleavage products, HPLC methods, and synthesis protocol for Fmoc-HfLeu-OH. [file Beilstein_J_Org_Chem-13-2869-s001.pdf]

Supporting Information

for

Position-dependent impact of hexafluoroleucine and trifluoroisoleucine on protease digestion

Susanne Huhmann[1], Anne-Katrin Stegemann[1], Kristin Folmert[1], Damian Klemczak[1], Johann Moschner[1], Michelle Kube[1] and Beate Koksch*[1]

Address: [1]Department of Chemistry and Biochemistry, Freie Universität Berlin, Takustraße 3, 14195 Berlin, Germany

Email: Beate Koksch - beate.koksch@fu-berlin.de

* Corresponding author

Characterization and identification of synthesized peptides, characterization of the enzymatic digestion reactions, and identification of proteolytic cleavage products, HPLC methods, and synthesis protocol for Fmoc-HfLeu-OH

Synthesis of Fmoc-HfLeu-OH

General information

All reactions were run under an argon atmosphere unless otherwise indicated. Room temperature refers to 22 °C. Reagents and anhydrous solvents were transferred *via* oven-dried syringe or cannula. Flasks were flame-dried under vacuum and cooled under a constant stream of argon. Reactions were monitored by thin layer chromatography using Merck KGaA silica gel 60 F_{254} TLC aluminium sheets and visualized with ceric ammonium molybdate, vanillin staining solution or potassium permanganate staining solution. Chromatographic purification was performed as flash chromatography on Macherey-Nagel GmbH & Co. KG silica gel 60 M, 0.04–0.063 mm, using a forced flow of eluent (method of Still). Concentration under reduced pressure was performed by rotary evaporation at 40 °C at the appropriate pressure. Yields refer to chromatographically purified and spectroscopically pure compounds. NMR measurements were recorded on a JEOL-ECX400 (operating at 400 MHz for ^{1}H NMR, 101 MHz for ^{13}C NMR and 376 MHz for ^{19}F NMR). Chemical shifts δ are reported in ppm with the solvent resonance as the internal standard. Coupling constants *J* are given in Hertz (Hz). Multiplicities are classified by the following abbreviations: s = singlet, d = doublet, t = triplet, q = quartet, br = broad or m = multiplet and combinations thereof. High resolution mass spectra were obtained on an Agilent ESI-ToF 6220 (Agilent Technologies, Santa Clara, CA, USA).

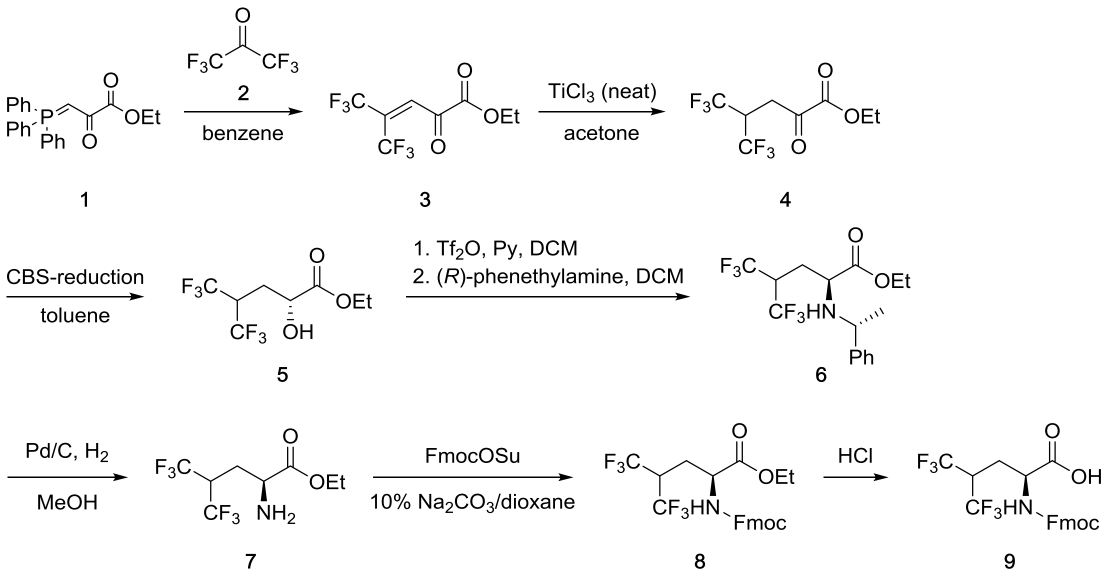

Scheme S1: Synthesis of Fmoc-HfLeu-OH **9**.

Compounds **3** to **7** were synthesized according to literature [1,2]. Obtained NMR data (^{1}H, ^{13}C, and ^{19}F) are consistent with literature [1,2].

<u>Synthesis of Fmoc-HfLeu-OEt (**8**)</u>

(*S*)-**7** (1.04 g, 3.89 mmol) was dissolved in 10% Na_2CO_3, aq (4 mL) and cooled to 0°C. Dioxane (1 mL) was added and the suspension was stirred for 15 min at 0 °C after which FmocOSu (1.44 g, 4.28 mmol) was added. The mixture was stirred for 3 h at 0 °C and at room temperature overnight. The reaction was diluted with H_2O (50 mL) and extracted with Et_2O (4 x 25 mL). The combined organic layers were concentrated in vacuo and the residue was subjected to column chromatography (*n*-hexane/Et_2O, 3:1) to give (*S*)-**8** (1.12 g, 2.29 mmol, 59%) as a waxy solid.

TLC: R_f = 0.45 (*n*-hexane/Et_2O, 5:1).

^{1}H-NMR (400 MHz, $CDCl_3$): δ = 7.77 (d, *J* = 7.5, 2H); 7.58 (d, *J* = 7.2, 2H); 7.40 (t, *J* = 7.5, 2H); 7.32 (t, *J* = 7.0, 2H); 5.40 (d, *J* = 7.50, 1H); 4.47 (dt, *J* = 20.0; 13.40, 3H); 4.32 – 4.18 (m, 3H); 3.18 (s, 1H); 2.40 (d, *J* = 14.5, 1H); 2.05 (d, *J* = 10.0, 1H); 1.30 (t, *J* = 7.1, 3H).

^{13}C-NMR (101 MHz, $CDCl_3$) δ = 170.68, 143.75, 143.53, 141.45, 141.44, 140.84, 130.32, 127.92, 127.90, 127.19, 127.17, 125.07, 124.98, 120.32, 120.15, 120.11, 67.29, 62.53, 51.96, 47.21, 37.15, 27.27, 14.13.

^{19}F-NMR (376 MHz, $CDCl_3$): δ = -67.27 – -67.44 (m), -67.63 – -67.79 (m).

HRMS calculated for $C_{23}H_{31}F_6NNaO_4$ $[M+Na]^+$: 512.1267; observed: 512.1294.

<u>Synthesis of Fmoc-HfLeu-OH (**9**)</u>

A solution of (*S*)-**8** (55.0 mg, 11.2 mmol) in HCl_{conc} (2 mL) was stirred at room temperature for 24 h. The crude product was lyophilized and purified via a LaPrepΣ low-pressure HPLC system (VWR, Darmstadt, Germany) using a Kinetex RP-C18 endcapped HPLC-column (5 µM, 100 Å, 250 × 21.2 mm, Phenomenex®, USA). Deionized water and acetonitrile (ACN), both containing 0.1% (v/v) TFA served as eluents. A linear gradient of 30–100% ACN + 0.1% (v/v) TFA over 18 min with a flow rate of 20.0 mL/min was applied. UV-detection occurred at 280 nm. This gave (*S*)-**9** (36.3 mg, 7.87 mmol, 70%) as a white powder.

^{1}H-NMR (400 MHz, DMSO-D_6): δ = 7.82 (d, *J* = 7.6, 2H); 7.77 (d, *J* = 8.7, 1H); 7.63 (d, *J* = 7.5, 2H); 7.36 (t, *J* = 7.4, 2H); 7.26 (t, *J* = 7.4, 2H); 4.35 – 4.23 (m, 2H); 4.17 (t, *J* = 6.7, 1H); 4.04 (br, 1H); 2.30 – 2.17 (m, 1H); 2.13 – 2.01 (m, 1H).

^{13}C-NMR (101 MHz, DMSO-D$_6$): δ = 175.95 (s); 158.87 (s); 144.17 (s); 144.10 (s); 141.23 (s); 141.22 (s); 128.27 (s); 128.25 (s); 127.62 (s); 127.61 (s); 125.69 (s); 125.69 (s); 125.63 (s); 125.61 (s); 120.65 (s); 120.61 (s); 66.23 (s); 51.54 (s); 47.11 (s); 29.52 (s); 26.34 (s).
^{19}F-NMR (376 MHz, DMSO-D6): δ = -65.91 – -66.13 (m); -66.38 – -66.62 (m).
HRMS calculated for $C_{21}H_{17}F_6NO_4$ $[M+Na]^+$: 484.0954; observed: 484.0942.

Peptide synthesis, purification and characterization

Peptide synthesis

Peptides containing HfLeu were synthesized on an Activo P11 Automated Peptide Synthesizer (Activotec, Cambridge, United Kingdom) working under nitrogen atmosphere. All other peptides, either non-fluorinated or TfIle containing, were synthesized manually under standard conditions.

Peptide characterization

High resolution mass spectra were recorded on an Agilent 6220 ESI–ToF LC–MS spectrometer (Agilent Technologies Inc., Santa Clara, CA, USA) to identify the pure peptide products. The samples were dissolved in a 1:1 mixture of water and acetonitrile containing 0.1% (v/v) TFA and injected directly into the spray chamber by a syringe pump using a flow rate of 10 µL min^{-1}. A spray voltage of 3.5 kV was used, the drying gas glow rate was set to 5 L min^{-1} and the nebulizer to 30 psi. The gas temperature was 300 °C.
To verify purity of the synthesized peptides analytical HPLC was carried out on a Chromaster 600 bar DAD-System with CSM software (VWR/Hitachi, Darmstadt, Germany). The system works with a low-pressure gradient containing a HPLC-pump (5160) with a 6-channel solvent degasser, an organizer, an autosampler (5260) with a 100 µL sample loop, a column oven (5310) and a diode array flow detector (5430). A LUNA™ C8 (2) column (5 µm, 250 × 4.6 mm, Phenomenex®, Torrance, CA, USA) was used. As eluents water and ACN, both containing 0.1% (v/v) TFA were used, the flow rate was adjusted to 1 mL/min and the column was heated to 24 °C. The used gradient method is shown in Table S1. The UV-detection of the peptides occurred at 220 nm. The data were analyzed with EZChrom Elite software (version 3.3.2, Agilent Technologies, Santa Clara, CA, USA).

Table S1: Used linear gradient for the purity determination of the synthesized peptides.

Time [min]	Water + 0.1% (v/v) TFA [%]	ACN + 0.1% (v/v) TFA [%]
0	95	5
18	30	70
19	0	100
21	0	100
21.5	95	5
24	95	5

Table S2: Identification of the synthesized peptides by ESI–ToF mass spectrometry and analytical RP-HPLC.

Peptide	Retention time [min]	Charge	m/z calculated	m/z observed
**FA**	10.597	+1	967.5364	967.5396
		+2	484.2721	484.2736
**P2-LeuFA**	12.500	+1	1009.5463	1009.5849
		+2	505.2956	505.2970
**P2-HfLeuFA**	12.393	+1	1117.4622	1117.5306
		+2	559.2573	559.2691
**P2-IleFA**	12.137	+1	1009.5463	1009.5849
		+2	505.2956	505.2971
**P2-TfIleFA**	12.493	+1	1063.4622	1063.5576
		+2	532.2814	532.2845
**P1'-LeuFA**	11.773	+1	1009.5463	1009.5863
		+2	505.2956	505.7982
**P1'-HfLeuFA**	11.917	+1	1117.4622	1117.5272
		+2	559.2573	559.2684
**P1'-IleFA**	11.370	+1	1009.5463	1009.5858
		+2	505.2956	505.2975
**P1'-TfIleFA**	11.870	+1	1063.4622	1063.5556
		+2	532.2814	532.2816
**P2'-LeuFA**	11.847	+1	1009.5463	1009.5866
		+2	505.2956	505.2981
**P2'-HfLeuFA**	12.197	+1	1117.4622	1117.5305
		+2	559.2573	559.2693
**P2'-IleFA**	11.557	+1	1009.5463	1009.5864
		+2	505.2956	505.2980
**P2'-TfIleFA**	12.283	+1	1063.4622	1063.5576
		+2	532.2814	532.2835

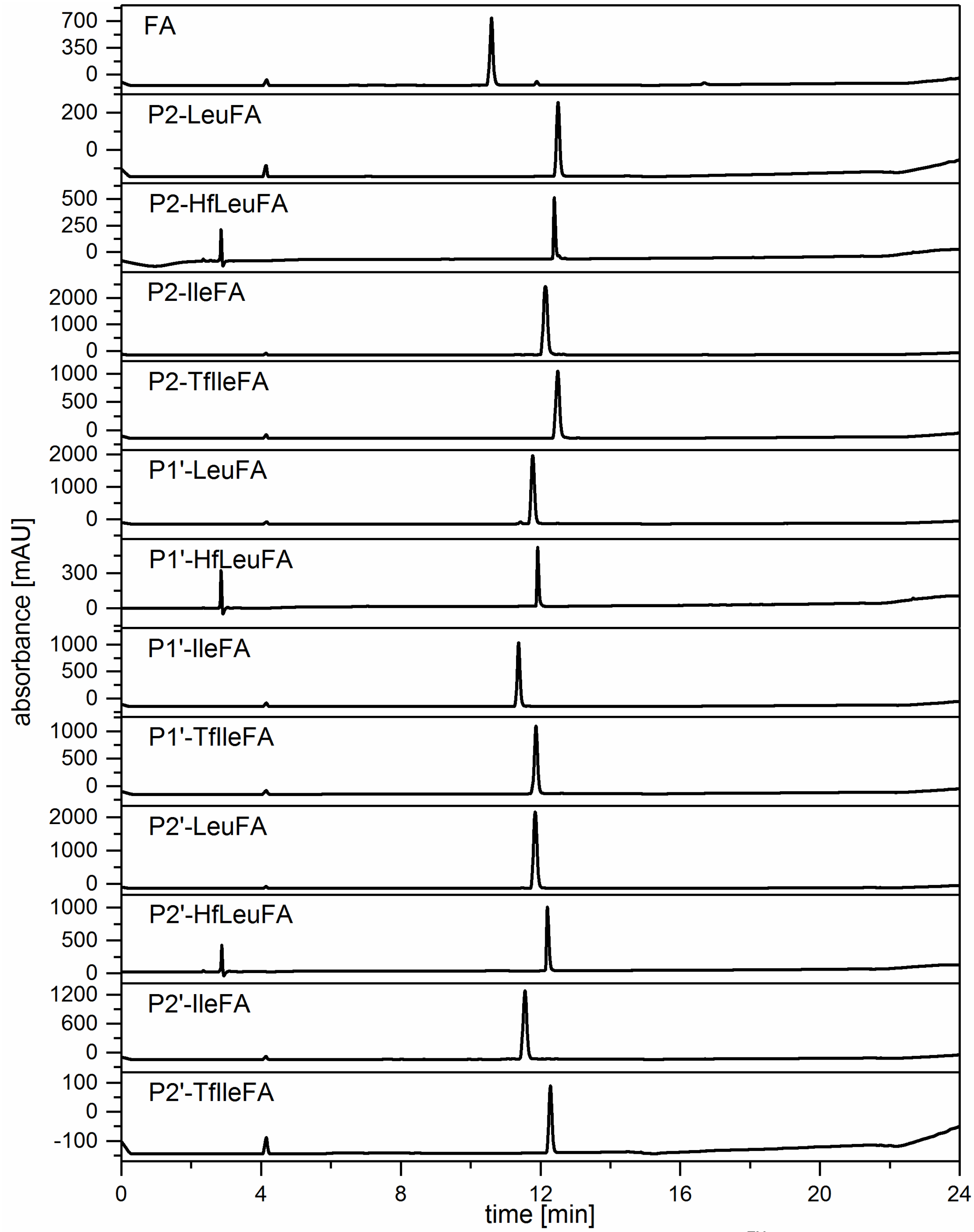

Figure S1: Analytical HPLC chromatograms of purified peptides; column: Luna^{TM}C8 (5 µM, 250 × 4.6 mm, Phenomenex®); Solvent A was H_2O, solvent B was acetonitrile, both containing 0.1% (v/v) TFA. The flow rate was 1 mL/min; linear gradient from 5% B to 70% B over 18 min (see Table S1).

Enzymatic digestion studies

Characterization of the enzymatic digestion reactions was carried out via analytical HPLC on a LaChrom-ELITE-HPLC-System from VWR International Hitachi (Darmstadt, Germany). The system contains an organizer, two HPLC-pumps (L-

2130) with solvent degasser, an autosampler (L-2200) with a 100 µL sample loop, a diode array flow detector (L-2455), a fluorescence detector (L-2485) and a high pressure gradient mixer. As eluents water and ACN, both containing 0.1% (v/v) TFA were used, and a flow rate of 3 mL/min was applied. The used linear gradients are shown in Table S3. For the non-fluorinated peptides method A was used to follow the digestion process, and for the fluorinated peptides method B was applied. For chromatograms where an insufficient baseline separation was observed, measurements were repeated using methods C [FA (pepsin), P2-LeuFA (proteinase K), P2-IleFA (pepsin), P2-IleFA (proteinase K), P1'-LeuFA (elastase), P1'-LeuFA (proteinase K), P1'-IleFA (proteinase K)] or D [P2-HfleuFA (proteinase K), P2-TfIleFA (pepsin), P2-TfIleFA (proteinase K), P1'-TfIleFA (elastase), P2'-TfIleFA (proteinase K)]. The obtained data were analyzed with EZChrom Elite software (version 3.3.2, Agilent Technologies, Santa Clara, CA, USA).

Table S3: Used linear gradients to follow the digestion process by FL-RP-HPLC.

Method	Time [min]	Water + 0.1% (v/v) TFA [%]	ACN + 0.1% (v/v) TFA [%]
**A**	0	95	5
	5	70	30
	5.5	70	30
	6	95	5
	9	95	5
**B**	0	95	5
	5	60	40
	5.5	60	40
	6	95	5
	9	95	5
**C**	0	95	5
	15	70	30
	15.5	70	30
	16	95	5
	17	95	5
**D**	0	95	5
	15	55	45
	15,5	55	45
	16	95	5
	17	95	5

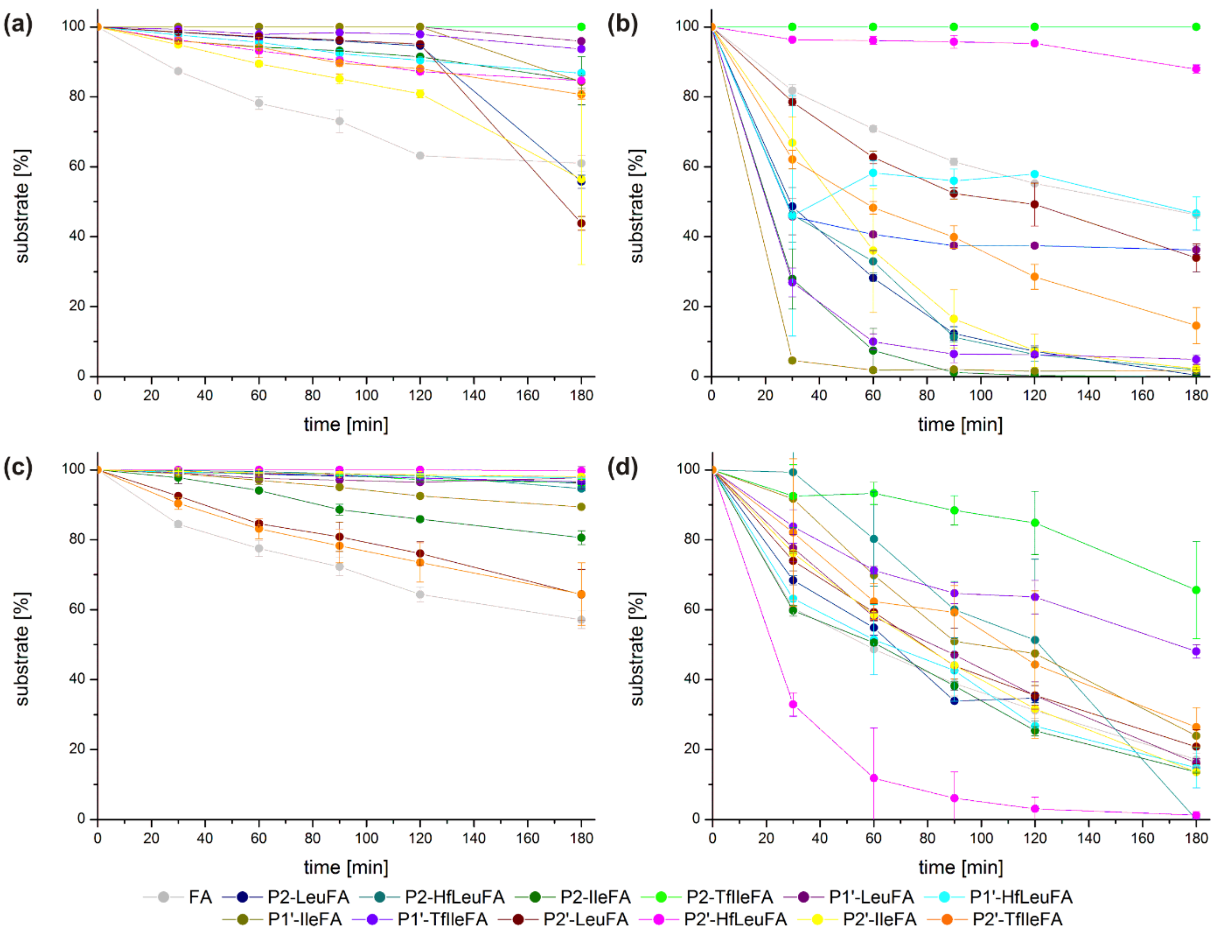

Figure S2: Chronological sequence of the substrate amount [%] over an incubation time of 180 min with **(a)** α-chymotrypsin, **(b)** pepsin, **(c)** elastase, and **(d)** proteinase K. The depicted values represent the mean of three independent measurements.

Identification of the proteolytic cleavage products (Table S4–S7) occurred according to the mass-to-charge ratios determined with an Agilent 6220 ESI–ToF–MS instrument (Agilent Technologies, Santa Clara, CA, USA). For this, the quenched peptide-enzyme-solutions after 120 min and 24 h incubation were analyzed. The solutions were injected directly into the spray chamber using a syringe pump with a flow rate of 10 µL min^{-1}. Spray voltage was set to 3.5 kV, a drying gas flow rate of 5 L min^{-1} was used, the nebulizer was set to 30 psi, and the gas temperature to 300 °C. The fragmentor voltage was 200 V. Not all corresponding fragments could be detected.

Table S4: Identification of the cleavage products of the different peptides by ESI–ToF mass spectrometry after digestion with α–chymotrypsin.

Peptide	Fragment	$[M + H]^{1+}$ calculated	$[M + H]^{1+}$ observed
**FA**	Abz-KAAFAAAAK	967.5364	967.5376
	Abz-KAAF	555.2559	555.2938
	AAAAK	431.2617	431.2627
**P2-LeuFA**	Abz-KALeuFAAAAK	1009.5463	1009.5883
	Abz-KALeuF	597.3029	597.2609

	AAAAK	431.2617	431.2617
**P2-HfLeuFA**	Abz-KAHfLeuFAAAAK	1117.4622	1117.5298
**P2-IleFA**	Abz-KAIleFAAAAK	1009.5463	1009.5851
	Abz-KAIleF	597.3029	597.3435
	AAAAK	431.2617	431.2647
**P2-TfIleFA**	Abz-KATfIleFAAAAK	1063.4622	1063.5577
**P1'-LeuFA**	Abz-KAAFLeuAAAK	1009.5463	1009.5866
	Abz-KAAFLeu	668.3400	668.3760
	Abz-KAAF	555.2559	555.2907
	LeuAAAK	473.3087	473.3087
	AAAK	360.2246	360.2239
**P1'-HfLeuFA**	Abz-KAAFHfLeuAAAK	1117.4622	1117.5280
	Abz-KAAFHfLeu	776.2559	776.3214
	HfLeuAAAK	581.2246	581.2246
	Abz-KAAF	555.2559	555.2934
	AAAK	360.2246	360.3630
**P1'-IleFA**	Abz-KAAFIleAAAK	1009.5463	1009.5825
	Abz-KAAF	555.2559	555.2951
	IleAAAK	473.3087	473.3104
**P1'-TfIleFA**	Abz-KAAFTfIleAAAK	1063.4622	1063.5604
	Abz-KAAF	555.2559	555.2954
	TfIleAAAK	527.2246	527.2827
**P2'-LeuFA**	Abz-KAAFALeuAAK	1009.5463	1009.5872
	Abz-KAAF	555.2559	555.2922
	ALeuAAK	473.3087	473.3087
**P2'-HfLeuFA**	Abz-KAAFAHfLeuAAK	1117.4622	1117.5331
	AHfLeuAAK	581.2246	581.2550
	Abz-KAAF	555.2559	555.2965
**P2'-IleFA**	Abz-KAAFAIleAAK	1009.5463	1009.5875
	Abz-KAAF	555.2559	555.2943
	AIleAAK	473.3087	473.3112
**P2'-TfIleFA**	Abz-KAAFATfIleAAK	1063.4622	1063.5575
	Abz-KAAF	555.2559	555.2945
	ATfIleAAK	527.2246	527.2822

Table S5: Identification of the cleavage products of the different peptides by ESI–ToF mass spectrometry after digestion with pepsin.

Peptide	Fragment	$[M + H]^{1+}$ calculated	$[M + H]^{1+}$ observed
**FA**	Abz-KAAFAAAAK	96.5364	967.5434
	Abz-KAAF	555.2559	555.2967
	AAAAK	431.2617	431.2617
**P2-LeuFA**	Abz-KALeuFAAAAK	1009.5463	1009.5895
	Abz-KALeuF	597.3029	597.3438
	AAAAK	431.2617	431.2642
**P2-HfLeuFA**	Abz-KAHfLeuFAAAAK	1117.4622	1117.5302
	Abz-KAHfLeuF	705.2188	705.2870
	FAAAAK	578.3301	578.3327
	AAAAK	431.2617	431.2636
**P2-IleFA**	Abz-KAIleFAAAAK	1009.5463	1009.5916
	Abz-KAIleF	597.3029	597.3442
	AAAAK	431.2617	431.2647
**P2-TfIleFA**	Abz-KATfIleFAAAAK	1063.4622	1063.5639
**P1'-LeuFA**	Abz-KAAFLeuAAAK	1009.5463	1009.5926

	Abz-KAAFLeu	668.3400	668.3820
	Abz-KAAF	555.2559	555.2971
	LeuAAAK	473.3087	473.3126
	AAAK	360.2246	360.2271
**P1'-HfLeuFA**	Abz-KAAFHfLeuAAAK	1117.4622	1117.5325
	Abz-KAAFHfLeu	776.2559	776.3236
	HfLeuAAAK	581.2246	581.2553
	Abz-KAAF	555.2559	555.2956
	AAAK	360.2246	360.2273
**P1'-IleFA**	Abz-KAAFIleAAAK	1009.5463	1009.5908
	Abz-KAAF	555.2559	555.2969
	IleAAAK	473.3087	437.3087
**P1'-TfIleFA**	Abz-KAAFTfIleAAAK	1063.4622	1063.5634
	Abz-KAAF	555.2559	555.2969
	TfIleAAAK	527.2246	527.2843
**P2'-LeuFA**	Abz-KAAFALeuAAK	1009.5463	1009.5905
	Abz-KAAF	555.2559	555.2963
	ALeuAAK	473.3087	473.3117
**P2'-HfLeuFA**	Abz-KAAFAHfLeuAAK	1117.4622	1117.5307
	Abz-KAAFA	626.2930	626.3344
	HfLeuAAK	510.1875	510.2170
**P2'-IleFA**	Abz-KAAFAIleAAK	1009.5463	1009.5889
	Abz-KAAF	555.2559	555.2970
	AIleAAK	473.3087	473.3121
**P2'-TfIleFA**	Abz-KAAFATfIleAAK	1063.4622	1063.5627
	FATfIleAAK	674.2930	674.3530
	Abz-KAAFA	626.2930	626.3333
	Abz-KAAF	555.2559	555.2969
	ATfIleAAK	527.2246	527.2845
	TfIleAAK	456.1875	456.2462

Table S6: Identification of the cleavage products of the different peptides by ESI–ToF mass spectrometry after digestion with elastase.

Peptide	Fragment	$[M + H]^{1+}$ calculated	$[M + H]^{1+}$ observed
**FA**	Abz-KAAFAAAAK	967.5364	967.5352
	Abz-KAAFAAA	768.3672	768.4080
	Abz-KAAFAA	697.3301	697.3690
	AFAAAAK	649.3673	648.2935
	Abz-KAAFA	626.2930	626.3308
	AAAK	360.2246	360.2238
	AAK	289.1875	289.1872
**P2-LeuFA**	Abz-KALeuFAAAAK	1009.5463	1009.5871
	Abz-KALeuFAAAA	881.4513	881.4831
	Abz-KALeuFAAA	810.4142	810.4831
	Abz-KALeuFAA	739.4116	739.4116
	LeuFAAAAK	691.4142	691.4116
	Abz-KALeuFA	668.3404	668.3745
	AAAAK	431.2617	430.0496
	AAAK	360.2246	360.2205
	AAK	289.1875	289.1858
**P2-HfLeuFA**	Abz-KAHfLeuFAAAAK	1117.4622	1117.5325
	Abz-KAHfLeuFAAA	918.3301	918.3980
	Abz-KAHfLeuFAA	847.2930	847.3610

	Abz-KAHfLeuFA	776.2559	776.2559
	FAAAAK	578.3301	578.2440
**P2-IleFA**	Abz-KAIleFAAAAK	1009.5463	1009.5884
	Abz-KAIleFAAAA	881.4513	881.4960
	Abz-KAIleFAAA	810.4142	810.4960
	Abz-KAIleFAA	739.3771	739.4176
	Abz-KAIleFA	668.3400	668.3787
	FAAAAK	578.3301	578.3322
	AAAK	360.2246	360.2256
	AAK	289.1875	289.1880
	AK	218.1504	218.1505
**P2-TfIleFA**	Abz-KATfIleFAAAAK	1063.4622	1063.5576
	Abz-KATfIleFAAA	864.3301	864.4210
	Abz-KATfIleFAA	793.2930	793.3844
	Abz-KATfIleFA	722.2559	722.3470
	FAAAAK	578.3301	578.3271
	AAAK	360.2246	360.224
**P1'-LeuFA**	Abz-KAAFLeuAAAK	1009.5463	1009.5887
	Abz-KAAFLeuAAA	881.4513	881.4815
	Abz-KAAFLeuAA	810.4142	810.4472
	AAFLeuAAAK	762.4513	762.4458
	Abz-KAAFLeuA	739.3771	7394120
	AFLeuAAAK	691.4142	691.4142
	FLeuAAAK	620.3771	620.3783
	Abz-KAA	408.1875	408.1875
	Abz-KA	337.1504	337.1504
	AAK	289.1504	289.1858
**P1'-HfLeuFA**	Abz-KAAFHfLeuAAAK	1117.4622	1117.5330
	Abz-KAAFHfLeuAA	918.3301	918.3982
	AAFHfLeuAAAK	870.3673	870.3975
	AFHfLeuAAAK	799.3301	799.3600
	Abz-KA	337.1504	337.1864
**P1'-IleFA**	Abz-KAAFIleAAAK	1009.5463	1009.5853
	Abz-KAAFIleAA	810.4142	810.4500
	Abz-KAAFIleA	739.3771	739.4144
	FIleAAAK	620.3771	620.3747
**P1'-TfIleFA**	Abz-KAAFTfIleAAAK	1063.4622	1063.5578
	Abz-KAAFTfIleAA	864.3301	864.4205
	Abz-KAAFTfIleA	793.2930	793.3844
	AFTfIleAAAK	745.3301	745.3814
	AAK	289.1875	289.1857
	AK	218.1504	218.1483
**P2'-LeuFA**	Abz-KAAFALeuAAK	1009.5463	1009.5894
	Abz-KAAFALeuAA	881.4513	881.4938
	Abz-KAAFALeuA	810.4142	810.4938
	AFALeuAAK	691.4142	691.4168
	Abz-KAAFA	626.2930	626.3324
	LeuAAK	402.2716	402.2730
	Abz-KA	337.1504	337.1872
**P2'-HfLeuFA**	Abz-KAAFAHfLeuAAK	1117.6622	1117.5330
	Abz-KAAFAHfLeuA	918.3301	918.4349
	AAFAHfLeuAAK	870.3673	870.4001
	AFAHfLeuAAK	799.3301	799.3612

	FAHfLeuAAK	728.2930	728.3229
	Abz-KAAFA	626.2930	626.3230
	Abz-KAAFA	408.1875	408.2262
**P2'-IleFA**	Abz-KAAFAIleAAK	1009.5463	1009.5866
	Abz-KAAFAIleAA	881.4513	881.4910
	Abz-KAAFAIleA	810.4142	810.4539
	Abz-KAAFAIle	739.3771	739.4171
	FAIleAAK	620.3771	620.3687
	Abz-KAA	408.1875	408.2252
**P2'-TfIleFA**	Abz-KAAFATfIleAAK	1063.4622	1063.5530
	Abz-KAAFATfIle	793.2930	793.3851
	FATfIleAAK	674.2930	674.3441
	Abz-KAA	408.1875	408.2233
	AAK	289.1875	289.1855

Table S7: Identification of the cleavage products of the different peptides by ESI–ToF mass spectrometry after digestion with proteinase K.

Peptide	Fragment	[M + H]$^{1+}$ calculated	[M + H]$^{1+}$ observed
**FA**	Abz-KAAFAAAAK	967.5364	967.5376
	AFAAAAK	649.3673	649.2762
**P2-LeuFA**	Abz-KALeuFAAAAK	1009.5463	1009.5863
	Abz-KALeuFAAA	810.4142	810.4536
	Abz-KALeuFAA	739.4116	739.4179
	Abz-KALeuFA	668.3404	668.3816
	Abz-KALeu	450.2345	450.2719
	AAK	289.1504	289.1880
**P2-HfLeuFA**	Abz-KAHfLeuFAAAAK	1117.4622	1117.5298
	Abz-KAHfLeuFAAA	918.3301	918.3970
	Abz-KAHfLeuFAA	847.2930	847.3608
	Abz-KAHfLeuFA	776.2559	776.3233
	Abz-KAHfLeu	558.1504	558.2168
	AAK	289.1875	289.1884
**P2-IleFA**	Abz-KAIleFAAAAK	1009.5463	1009.5872
	Abz-KAIleFAAA	810.4142	810.4551
	Abz-KAIleFAA	739.3771	739.4192
	Abz-KAIleFA	668.3400	889.3806
	Abz-KAIleF	597.3029	597.3422
	AAK	289.1875	289.1891
**P2-TfIleFA**	Abz-KATfIleFAAAAK	1063.4622	1063.5604
	Abz-KATfIleFAAA	864.3301	864.4204
	Abz-KATfIleFAA	793.2930	793.3895
	Abz-KATfIleFA	722.2559	722.3512
	Abz-KATfIleF	651.2188	651.3140
	AAAAK	431.2617	430.0513
	AAK	289.1875	289.1888
**P1'-LeuFA**	Abz-KAAFLeuAAAK	1009.5463	1009.5877
	Abz-KAAFLeuAA	810.4142	810.4552
	Abz-KAAFLeuA	739.3771	739.4182
	FLeuAAAK	620.3771	620.3813
	Abz-KAA	408.1875	408.2276
	AAK	289.1504	2891885
**P1'-HfLeuFA**	Abz-KAAFHfLeuAAAK	1117.4622	1117.5271
	FHfLeuAAAK	728.2930	728.3226

	Abz-KAA	408.1875	408.2261
**P1'-IleFA**	Abz-KAAFIleAAAK	1009.5463	1009.5878
	Abz-KAAFIleAA	810.4142	810.4545
	Abz-KAAFIleA	739.3771	739.4169
	FIleAAAK	620.3771	620.3795
	Abz-KAA	408.1875	408.2265
	AAK	289.1504	289.1891
**P1'-TfIleFA**	Abz-KAAFTfIleAAAK	1063.4622	1063.5580
	Abz-KAAFTfIleAA	864.3301	864.4253
	Abz-KAAFTfIleA	793.2930	793.3853
	FTfIleAAAK	647.2930	674.3504
	Abz-KAA	408.1875	408.2260
	AAK	289.1875	289.1880
	AK	218.1504	218.1508
**P2'-LeuFA**	Abz-KAAFALeuAAK	1009.5463	1009.5845
	Abz-KAAFALeu	739.3771	739.4166
	FALeuAAK	620.3771	620.3782
	Abz-KAA	408.1875	408.2252
	AAK	289.1875	289.1883
**P2'-HfLeuFA**	Abz-KAAFAHfLeuAAK	1117.4622	1117.5304
	Abz-KAAFAHfLeu	847.2930	847.3603
	HfLeuAAK	510.1875	510.2172
	AAK	289.1875	268.1885
**P2'-IleFA**	Abz-KAAFAIleAAK	1009.5463	1009.5854
	Abz-KAAFA	626.2930	626.338
	FAIleAAK	620.3771	620.3791
	Abz-KAA	408.1875	408.2260
**P2'-TfIleFA**	Abz-KAAFATfIleAAK	1063.4622	1063.5563
	FATfIleAAK	674.2930	674.3496
	Abz-KAA	408.1875	408.2239

Reference

1. Chiu, H.-P.; Cheng, R. P. *Org. Lett.* **2007,** *9* (26), 5517-5520.
2. Zhang, C.; Ludin, C.; Eberle, M. K.; Stoeckli-Evans, H.; Keese, R. *Helv. Chim. Acta* **1998,** *81* (1), 174-181.
